# Thermodynamic Swings: How Ideal Complex of Cas9–RNA/DNA Forms

**DOI:** 10.3390/ijms23168891

**Published:** 2022-08-10

**Authors:** Polina V. Zhdanova, Alexander A. Lomzov, Daria V. Prokhorova, Grigory A. Stepanov, Alexander A. Chernonosov, Vladimir V. Koval

**Affiliations:** 1 Institute of Chemical Biology and Fundamental Medicine, Siberian Branch of the Russian Academy of Sciences (SB RAS), 630090 Novosibirsk, Russia; 2 Department of Natural Sciences, Novosibirsk State University, 630090 Novosibirsk, Russia

**Keywords:** CRISPR–Cas systems, Cas9, single-guide RNA, molecular dynamics, ITC, thermodynamics

## Abstract

Most processes of the recognition and formation of specific complexes in living systems begin with collisions in solutions or quasi-solutions. Then, the thermodynamic regulation of complex formation and fine tuning of complexes come into play. Precise regulation is very important in all cellular processes, including genome editing using the CRISPR–Cas9 tool. The Cas9 endonuclease is an essential component of the CRISPR–Cas-based genome editing systems. The attainment of high-specificity and -efficiency Cas9 during targeted DNA cleavage is the main problem that limits the practical application of the CRISPR–Cas9 system. In this study, we analyzed the thermodynamics of interaction of a complex’s components of Cas9–RNA/DNA through experimental and computer simulation methods. We found that there is a small energetic preference during Cas9–RNA/DNA formation from the Cas9–RNA and DNA/DNA duplex. The small difference in binding energy is relevant for biological interactions and could be part of the sequence-specific recognition of double-stranded DNA by the CRISPR–Cas9 system.

## 1. Introduction

CRISPR (Clustered regularly interspaced short palindromic repeats)–Cas (CRISPR-associated proteins) systems offer bacteria-adaptive protection against extraneous nucleic acids [1,2]. CRISPR–Cas systems vary [3]; however, at present, the type II systems, which are defined by the Cas9 nuclease, are most widely used as a genome-editing toolbox [4]. However, these systems nevertheless suffer from a systemic disadvantage: it cuts completely and partially complementary DNA [5,6,7]. Along with the Cas9 protein, the CRISPR–Cas9 system consists of a single-stranded RNA guide (sgRNA), which is a hybrid chimeric form of the crRNA complex and tracrRNA [8]. Such a system tolerates up to six mismatches within the sgRNA’s complementary region of transfer DNA (tDNA) [8,9]. Cas9 also cleaves double-stranded target DNA (tDNA) in the absence of the protospacer adjacent motif sequence in dsDNA (dsDNA) [4,10]. Common mistakes include insertions and deletions in tDNA in the tDNA/sgRNA complementary region [11].

To improve the accuracy of Cas9 as a genomic editing tool, more detailed studies of protein structure, protein–ligand and protein–protein interactions are required. The current method for determining the spatial structure is to obtain the crystal structure of the protein [12], but this does not provide information on the interaction with RNA and DNA. For molecular modeling of protein complexes [13,14] or to study their structure in solution, mass spectrometry using hydrogen–deuterium exchange [15,16] was used to analyze protein–ligand and protein–protein interactions.

Selecting the target region of the genome is the first step when using CRISPR–Cas9. Therefore, an important issue is the selection of a gRNA sequence with different cleavage efficiencies at the target site. The molecular mechanisms of recognition specificity and cleavage efficiency still remain incompletely understood. One of the major problems limiting the in vivo applicability of CRISPR–Cas9 systems is off-target DNA cleavage. To understand the molecular basis of off-target binding, extensive experiments have been performed [17,18,19,20], including analysis of the solution structure of Cas9 from *S. pyogenes* using hydrogen–deuterium exchange mass spectrometry [21]. However, fundamental characterization of the thermodynamic aspects of this process is necessary to better explain the interaction of CRISPR–Cas9 with the nucleic acid duplex. A major contribution to the study of off-target processes was made by Alkan and colleagues [22]. They proposed a method for estimating off-target sequences and specificity. This method allows the free energy of RNA/DNA duplex formation to be calculated for both off-target and target sequences. Along with computer modeling of CRISPR–Cas9 activity [23], actual measurement of the interaction energy is possible through isothermal titration calorimetry (ITC) [24,25,26,27,28]. ITC has already been applied for investigation of aptamer binding [29], molecular organization of CRISPR adaptation module [30], anti-CRISPR-mediated suppression mechanisms [31] and the interaction of Cas9 with inhibitors [32]. To date, full characterization of the thermodynamic parameters of Cas9 functioning remains an unresolved problem.

Here, we report the thermodynamic analysis of interactions accompanying Cas9–RNA/ssDNA complex formation performed by ITC experiments and by computer simulation. From the processing of the experimental data, we obtained the thermodynamic characteristics of the formation of the Cas9–RNA/DNA complex. The data allow us to reasonably conclude that small differences in ΔG for the fully complementary complex and the duplex containing a single nucleotide substitution are the drivers for discrimination of the correct/incorrect complex by Cas9 nuclease. Our data show the physicochemical basis for DNA processing by Cas9 and opens up perspectives for fine tuning of the CRISPR–Cas9 system.

## 2. Results and Discussion

The thermodynamics of the Cas9–RNA complex interaction with double-stranded DNA is important for understanding the specificity of CRISPR–Cas9. Alkan and colleagues in their study [22] developed methods to evaluate off-target effects and specificity based on calculations of the free energy of nucleic acid duplexes. This approach is valuable for predicting the intra- and intermolecular interactions of molecules, but it does not take into account the existence of the interface between the protein molecule and nucleic acids. In this paper, we performed ITC experiments, as well as molecular dynamics simulations, for complexes and their components. This approach makes it possible to not only evaluate the effect of nucleic acid interactions, but also understand the enzyme’s contribution to the processes of recognition, formation of the catalytically active complex, and cleavage. 

**Figure 1:**
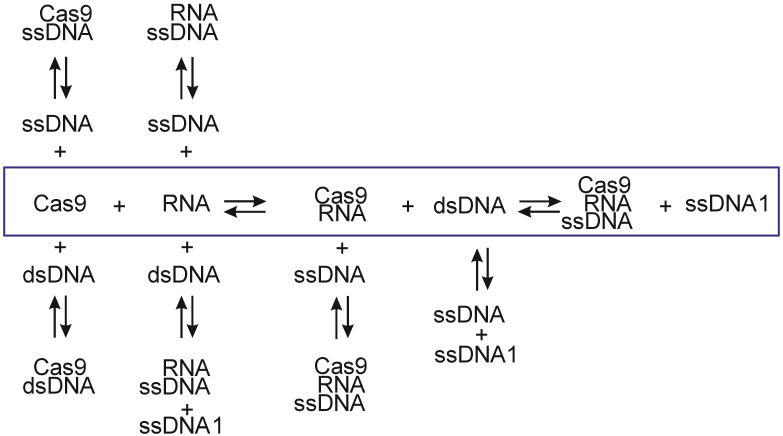
The schematic diagram of the possible interactions during formation of the Cas9–RNA/ssDNA complex.

A schematic diagram of the possible processes accompanying Cas9–RNA/ssDNA complex formation in vitro is presented in Figure 1. Here, we analyzed reversible processes at thermodynamic equilibrium. The main process is the interaction of Cas9 with RNA complex and, following the interaction with dsDNA, possessive complex Cas9–RNA/ssDNA formation (marked by an outline in Figure 1). Alternatively, the targeted complex can be formed by Cas9–RNA interaction with ssDNA. In addition to the main process of formation of the targeted complex, side reactions may occur with the formation of additional complexes. Free ssDNA can form complementary complexes with RNA and ssDNA1, or ssDNA1 can be displaced from dsDNA by free RNA. Moreover, non-specific interaction can occur by the interaction of Cas9 with single- or double-stranded DNA.

Therefore, we studied all the interactions presented in Figure 1, using isothermal calorimetry and molecular dynamics to determine the thermodynamic parameters of the interaction in the Cas9 system, guide RNA and DNA substrate.

### 2.1. ITC Experiments

**Figure 2:**
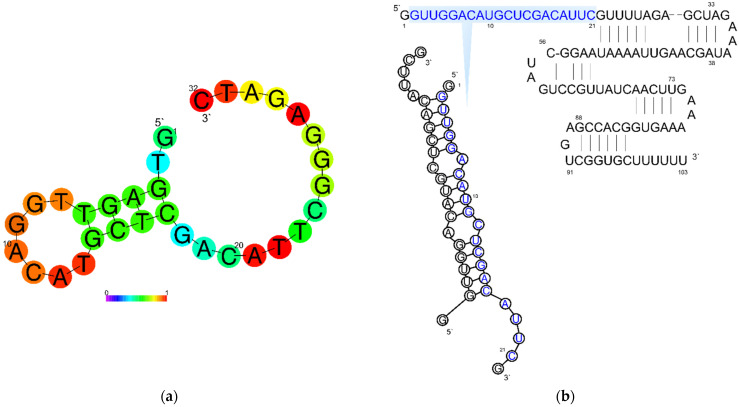
The possible secondary structure of ssDNA1 (**a**) and guide RNA (**b**) obtained by RNAFold webserver [38]. The gradient scale at the bottom shows the probability of base pair formation: blue color—the lowest probability (0); red color—the highest (1).

In the first step, the interaction of Cas9 with RNA was studied in order to find their concentrations for effective Cas9–RNA complex formation and further analysis. Furthermore, for the correct interpretation of the obtained thermodynamic parameters, it is important to analyze the possible interaction of components of the system. For this purpose, we analyzed the dissociation of possible self-complementary complexes by determining the thermal effects of RNA, ssDNA, and dsDNA dilution in buffer. Dilution of every studied nucleic acid into the solution did not lead to transition in the titration curves. The absence of thermal effects for dsDNA means that the formed duplex is sufficiently stable and does not dissociate upon dilution. In the case of dilution of RNA and ssDNA1, significant thermal effects are observed without transitions. This could be caused by the rearrangement of the intramolecular structure or dissociation of the RNA/RNA dimer. To determine the formation of intermolecular nucleic acid complexes—ssDNA/ssDNA1 (or dsDNA) and ssDNA/RNA—their components were mixed. In both cases, thermal effects were observed. A well-defined transition is found at the dsDNA formation, with a binding constant of 0.2 μM, an enthalpy of complexation of −212 kcal/mol and, close to unity, a chain interaction stoichiometry (*n*) of 0.83 corresponding to bimolecular complex formation. The difference of *n* from the stoichiometric value equal to 1 could be caused by the 20% accuracy error of the spectrophotometric method used in the work for oligonucleotide concentration measurement [33,34]. The obtained values of the enthalpy and entropy of complex formation are close to those calculated by the nearest neighbor model: −246 kcal/mol and −675 kcal/mol·K, respectively [35,36]. The calculated value of the melting temperature under the experimental conditions is about 79 °C. This suggests high stability of the dsDNA duplex and the impossibility of its dissociation during titration into the buffer at 25 °C, which agrees with previous dissociation experiments. Analysis of secondary structures using the RNAFold webserver (accessed on 16 April 2021 http://rna.tbi.univie.ac.at) [37] revealed no intramolecular structures in the ssDNA, and the presence of a relatively stable helix in complementary ssDNA1 (Figure 2). This could also partially explain the difference between the observed stoichiometry of 0.83 from 1.

In the study of the interaction of ssDNA with RNA, the thermal effects corresponding to the interaction are observed only at the first injections. Thus, it is impossible to reliably determine the thermodynamic parameters of complex formation due to the high measurement error. The thermodynamic parameters obtained by ITC using the independent binding model are summarized in Table 1. The calculated thermodynamic parameters using the nearest neighbor model [39], including the correction for ionic strength of the solution [40], are ΔH° at −280.1 kcal/mol, ΔS° at −763.5 kcal/mol/K and melting temperature at 73.0 °C.

The calculated ΔH° and ΔS° values differ significantly from the experimental results. This difference can be explained by the formation of secondary structures by the RNA chain in the DNA binding region. RNA structuring was confirmed by analyzing the secondary structure using the RNAFold webserver (Figure 2). Most of the RNA molecules are structured and are not available for interaction with DNA, resulting in an interaction stoichiometry (*n*) close to 0.1, i.e., only 10% of the RNA is able to interact with DNA. In addition to the inability of all RNA molecules to interact with DNA, the rearrangement of the intramolecular RNA complex into a conformation capable of interacting with RNA requires additional energy consumption. As a result, the ΔH° value observed at *n* = 0.1 is significantly different from the expected one. Nevertheless, the value of the dissociation constant at 0.64 μM indicates the formation of a stable complex, which correlates with the calculated melting temperature. 

Therefore, the formation of a stable DNA/DNA duplex and RNA stable secondary structure formation resulted in the absence of thermal effects for the interaction of RNA with dsDNA by ITC measurement (Figure S1e,f).

To determine the optimal concentrations of RNA and Cas9 for titration in the subsequent study on the main binding process by the Cas9–RNA complex with DNA (Figure 1), the RNA at 62.5 μM concentration was titrated into 5 μM of Cas9 solution. It was found that they form a stable complex with an ΔH° value of −399 kcal/mol, a binding constant at the micromolar level and Cas9 to RNA stoichiometry as 1:2 (Table 1). The parameter values were obtained in the independent binding model approximation.

The significant difference in the stoichiometry of the Cas9–RNA complex from unity also indicates that the RNA forms intramolecular complexes as shown above, which results in the prevention of protein binding. This highly enthalpic binding with non-unity could be also explained by Cas9 interaction with the RNA dimer. The RNA/RNA dissociation was found in ITC experiments of RNA dilution in buffer. Based on these data, we chose the stoichiometry of Cas9–RNA as 5/1 to be sure that all RNA molecules were in complex with the protein, to study the interaction of Cas9–RNA with DNA. This was required to prevent the free form of RNA binding to the DNA. However, with this excess of protein, free Cas9 can interact with ssDNA or dsDNA, which could affect the studied thermodynamic parameters. Therefore, we tested the possibility of Cas9 interacting with ssDNA or dsDNA and showed no interaction of Cas9 with ssDNA and dsDNA (Table 1, Figure S1g–j).

**Figure 3:**
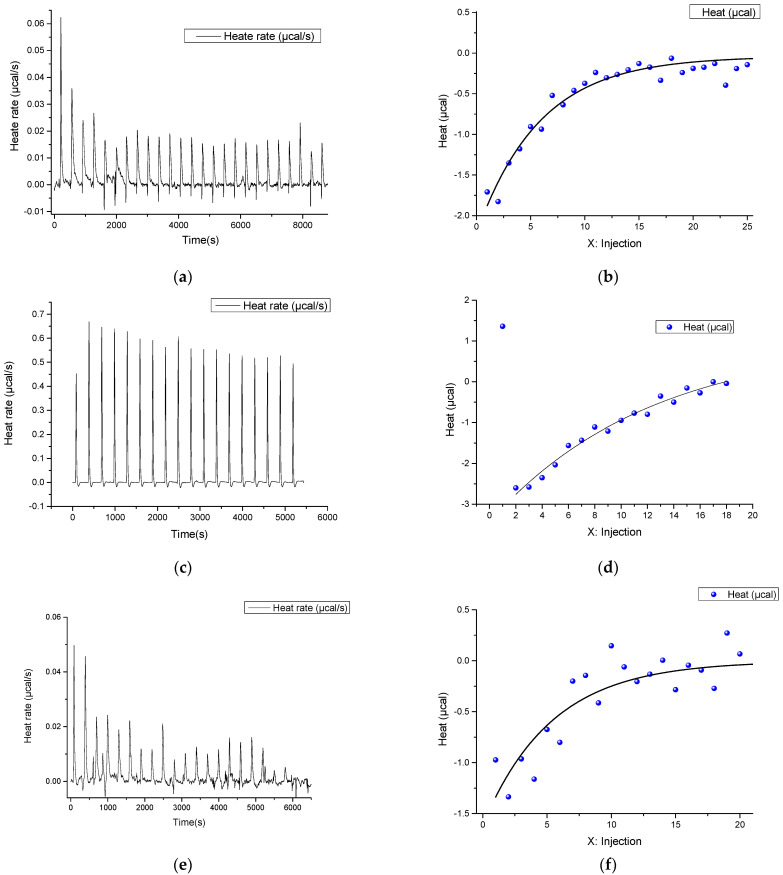
The ITC profiles (**a**,**c**,**e**) and corresponding integrated areas of each heat burst curve plotted as a function of injection number (**b**,**d**,**f**) for interaction of RNA with ssDNA (**a**,**b**), Cas9 with RNA (**c**,**d**) and Cas9–RNA complex with dsDNA (**e**,**f**).

Afterwards, taking into account the data on the possible interaction of all components, the formation of the Cas9–RNA complex with dsDNA was studied via ITC (Figure 3e,f). Small thermal effects were observed at the first injections. This was verified by carrying out a number of repeated experiments and experiments at different concentrations of dsDNA. The obtained results demonstrate the low interaction energy associated with the close dissociation energy of dsDNA and the energy of formation of the DNA/RNA duplex, which was calculated using the nearest neighbor method. The difference in enthalpy values of dsDNA and DNA/RNA complex formation is +35 kcal/mol, which correspond to the more profitable formation of the DNA/RNA duplex. This agrees well with the enthalpy value obtained experimentally (+45 kcal/mol). 

To determine the thermodynamic effects more reliably, we examined the interaction of the Cas9–RNA complex with ssDNA. The values obtained were ΔH° at −49.27 kcal/mol, ΔS° at −128.6 kcal/mol·K and ΔG° −10.93 kcal/mol, which are comparable to the parameters obtained in the interaction of Cas9–RNA with dsDNA. This could also suggest that Cas9 interacts with the RNA dimer in the absence of DNA.

Based on the interaction energies of the components of the complexes, one can judge such characteristics as, for example, specificity and selectivity. The obtained small differences in ΔG are the drivers for the determination of the correct/incorrect complex by the Cas9 nuclease.

### 2.2. Computer Simulations

To verify the experimental data, we performed computer simulations of the Cas9–RNA/DNA and Cas9–RNA complexes, the native form of Cas9, as well as RNA/DNA and dsDNA duplexes, and RNA and ssDNA separately using molecular dynamics. The obtained MD trajectories were analyzed by MMPB (GB)SA methods in order to calculate the thermodynamic parameters of the interaction between the components of the studied system (Figure 1) and subsequent comparison with the experimental results.

Molecular dynamics (MD) simulations for Cas9–RNA/DNA and Cas9–RNA complexes, the Cas9 protein, RNA/DNA and dsDNA duplexes began with the crystallographic structure of the Cas9–RNA/DNA complex (PDB ID: 4OO8), as described in the methods section. The whole structure of Cas9 was dynamic along the MD trajectory, which was evidenced by the RMSD values of the protein (Figure S2a). High values of RMSD corresponded to the movement of individual domains of the protein, whereas the domains themselves appeared to be rather conformationally stable.

The Cα-based RMSD calculations showed that the structure of the enzyme in the complex with RNA remains relatively stable starting from ~10 ns, and the deviation values lie in the range of 5–5.5 Å (Figure S2b). For the Cas9–RNA/DNA complex, the equilibration reached the first few nanoseconds of the molecular dynamics trajectory (RMSD values ~3 Å), and then the RMSD increased up to ~5 Å at ~70 ns (Figure S2c). 

**Figure 4:**
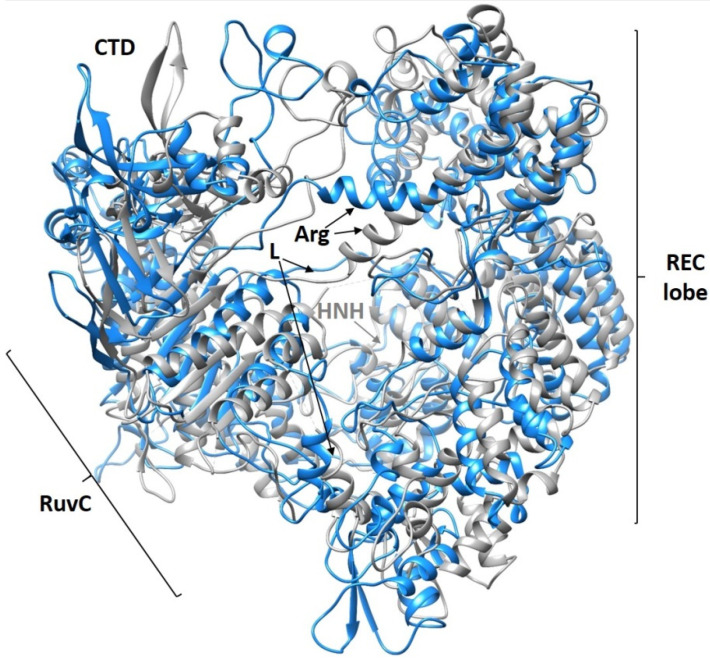
Comparison of the most represented Cas9 cluster (blue) with the structure obtained by X-ray crystallography PDB ID: 4OO8 (gray).

To determine the conformations most represented in the trajectory, we performed a cluster analysis of the molecular dynamics trajectories. The representation of clusters as a function of dynamics time is shown in Figure S3. The most significant difference is observed between the location of some domains in the structure of the protein most represented in the MD trajectory and the initial structure from the PDB database (PDB ID: 4OO8) (Figure 4). The domain structure of the protein does not change dramatically; only some minor rearrangements occur. According to the published data, the HNH domain is moved by 65 Å relative to the REC3 domain when the Cas9–RNA complex is formed [3]. We do not observe the reverse process of the divergence of these two domains in the 100 ns dynamics. However, when the protein complexes with nucleic acids form, the REC domain stabilizes, as can be seen from these RMSD plots. Considerable conformational changes in the REC1–3 domains allow sensing of nucleic acid adjustment and regulation of the HNH conformational transition, which finally leads to the formation of catalytically active CRISPR–Cas9 [20]. According to the RMSD plots, the RuvC and L-I,II domains undergo the greatest change (Figure S2b). The Cas9 RuvC domain cleaves the non-complementary strand of the target DNA by the two-metal mechanism. The L-II linker connects the HNH and RuvC domains and is labile in the apo-enzyme structure. We observe a deviation of the linker in the most represented structure from its position in the crystal structure. The last are partially loops and fluctuate freely in space in the absence of additional stabilizing factors. The Arg bridge significantly changes its conformation: in the Cas9–RNA complex, it remains rather rigid (straight), whereas in the apo-form of enzyme, it bends. The stabilization of the Arg bridge in protein–RNA complexes is also confirmed by RMSD plots (Figure S2a–c). The bridge helix is a universal structural feature of Cas9 proteins [12] and includes eight arginine residues (Arg−63, 66, 69, 70, 71, 74, 75, 78) that contact the sgRNA phosphate backbone.

Along with the protein analysis, the trajectories containing nucleic acids were analyzed for RNA structure flexibility. The RNA from the original complex was taken as a starting point for the simulation. The RNA molecule contains five self-complementary sites (four loops). RNA in the unbound state, i.e., without protein and DNA, fluctuates freely in space, but the hairpin structure remains stable. When the RNA/DNA complex is formed, the RNA at the complementarity site with DNA is stabilized by complementary interactions, although the entire molecule remains highly mobile. As expected in the complex with Cas9, the RNA molecule slightly deviates from its initial position, which is due to tight RNA–enzyme interactions.

To estimate the thermodynamic parameters of the formation of Cas9–RNA/DNA, Cas9–RNA, RNA/DNA, and dsDNA complexes, the free binding energies were calculated using the MM-PBSA (PB) and MM-GBSA (GB) methods (Table 2). To obtain the average values of the free binding energies, all snapshots of MD trajectory were analyzed. Since the RNA in the simulations without Cas9 had high flexibility, the MD analysis gives highly erroneous values during energy analysis. 

When we analyzed the formation of the Cas9–RNA/DNA complex, the trajectories of the complex components were isolated from the trajectory of the initial system, and the binding energy of the complex components was calculated. It amounted to ΔE = −363.9 ± 0.2 and ΔE = −149.0 ± 0.2 kcal/mol for the MM-GBSA and MM-PBSA methods, respectively. In the case of Cas9–RNA complex formation, we used component trajectories derived from the trajectory of the complex itself for thermodynamic analysis. In this case, the energy values are ΔE = −820.0 ± 0.3 (GB) and ΔE = −948.2 ± 0.4 (PB) kcal/mol.

It is important to note that the MM-GBSA method for calculating the energies of nucleic acid interaction has an error rate of ~9%, whereas MM-PBSA can give a significant errors in absolute values of enthalpy changes [41]. Despite this, the binding energies calculated by the MM-PBSA and MM-GBSA methods highly correlate, with R^2^ = 0.99 (Figure S4). This factor provides the main difference between the values for complexes, where the interaction of two nucleic acids was studied: Cas9–RNA/DNA, RNA/DNA, and dsDNA. The ΔE values for RNA/DNA and DNA/DNA complexes obtained by the MM-GBSA method are practically equal (−208.7 and −221.3 kcal/mol, respectively). Meanwhile, the difference in ΔE values for Cas9–RNA/DNA (−363.9 kcal/mol) and RNA/DNA (−208.7 kcal/mol) complexes may be caused by the contribution of the interaction of Cas9 with DNA. Indeed, in the Cas9–RNA/DNA complex, the DNA substrate is surrounded by the CTD, RuvC III, and REC2 and three domains.

**Figure 5:**
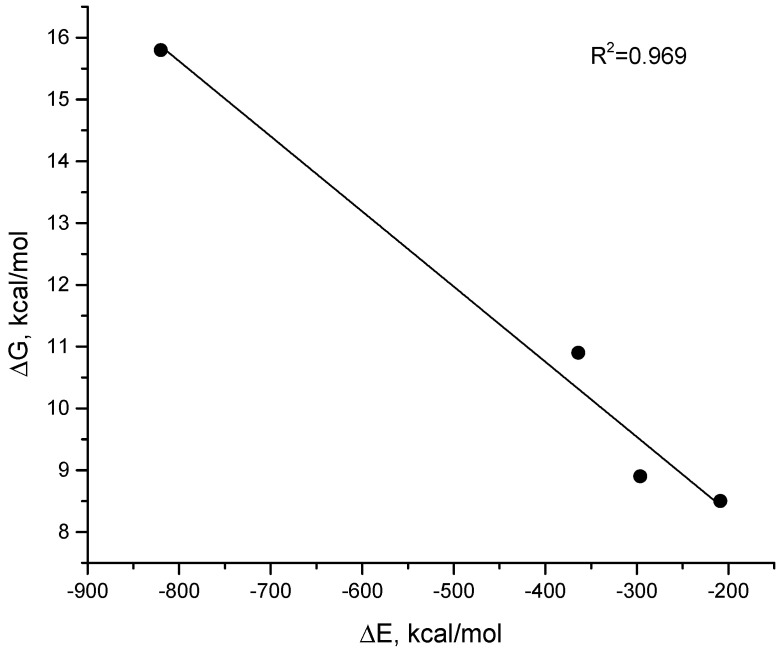
Correlation of the data obtained experimentally (ITC) and by MD simulations analysis (MM-GBSA). The *x*-axis shows the energies resulting from the MD analysis of the complex trajectories in kcal/mol, and the *y*-axis shows the ΔG from the ITC experiments, in kcal/mol.

Comparison of the theoretical calculations and calorimetry data showed good correlation of the obtained energy values (Figure 5). MM-GBSA values (since the estimate for all types of complexes is reasonable) and Gibbs energy from the experiment were used as comparison data. It should be noted that, in the case of Cas9–RNA/DNA, we took into account the processes of breaking the RNA/RNA dimer during the formation of the complex.

## 3. Materials and Methods

### 3.1. Preparation of Cas9 Enzyme

Plasmid encoding a SpyCas9, which contains an N-terminal 6x-Histidine tag, MBP sequence and TEV site (pMJ806), was obtained from Addgene (Watertown, MA, USA) (39312). Cas9 protein was prepared and purified similar to the previously described protocol [42].

In brief, protein expression was induced in Rosetta 2 DE3 cells (Novagen, Merck Millipore, Burlington, MA, USA) with 0.2 mM IPTG at 18 °C for 16 h. Cell pellets were resuspended in 15 mL of chilled lysis buffer (20 mM Tris-Cl, pH 8.0, 250 mM NaCl, 5 mM imidazole, pH 8.0, 1 mM phenylmethylsulfonyl fluoride) per cell pellet from 1 L culture. Resuspended cells were homogenized and clarified by centrifugation. His-Select Ni resin (Sigma-Aldrich, St. Louis, MO, USA) was equilibrated with lysis buffer. The cleared lysate was loaded onto the column, attached to an FPLC system, and equilibrated with wash buffer (20 mM Tris-Cl, pH 8.0, 250 mM NaCl, 10 mM imidazole, pH 8.0) at 4 °C. The supernatant was washed in 50 mL volumes of wash buffer. Protein was eluted with 50 mL of elution buffer (20 mM Tris-Cl, pH 8.0, 250 mM NaCl, 250 mM imidazole, pH 8.0). Furthermore, 0.5 mg of TEV protease was added per 50 mg of protein and the sample was dialyzed against 2 L of dialysis buffer (20 mM HEPES-KOH, pH 7.5, 150 mM KCl, 10% (*v*/*v*) glycerol, 1 mM dithiothreitol (DTT), 1 mM EDTA) at 4 °C overnight. The eluent of SpyCas9 was further purified over a HiTrap SP FF (GE Healthcare, Chicago, IL, USA) column equilibrated with IEX buffer B (20 mM HEPES-KOH, pH 7.5, 1 M KCl). Then, IEX buffer B was changed to the SEC buffer (20 mM HEPES-KOH, pH 7.5, 500 mM KCl, 1 mM DTT). Purified Cas9 enzyme was concentrated with an MWCO centrifugal concentrator (Sartorius, Göttingen, Germany, 30 K MWCO). Concentration was determined by UV absorbance at 280 nm.

### 3.2. Synthesis of Single-Guide RNA

The sgRNA was obtained by T7 transcription with DNA templates synthesized by PCR from plasmid pSpCas9(BB)-2A-GFP (PX458, Addgene #48138) with corresponding protospacer. The DNA template contained the T7 promoter to generate double-stranded promoter regions, which support in vitro transcription by T7 RNA polymerase [43]. In vitro transcription was performed according to the standard protocol overnight at 37 °C. The reaction mixture contained T7 RNA polymerase, 200 mM Tris-HCl (at pH 7.9), 30 mM MgCl2, 50 mM NaCl, 10 mM spermidine,10 mM DTT, 80 e.a. RiboLock RNAse inhibitor and 300 ng DNA template. Then, the DNA template was degraded by the addition of 5 units of DNase I for every 100 μL of reaction and incubated at 37 °C for 30 min. The RNA was purificated using phenol-chloroform extraction method followed by isolation on absorption columns using the LRU-100-50 kit (Biolabmix, Novosibirsk, Russia) or analogical mirVana™ miRNA isolation kit (Thermo Scientific, Waltham, MA, USA), and diluted with nuclease free water. Purified RNA was analyzed by gel electrophoresis and was quantified by measuring absorbance at 260 nm. The sequences of all the DNA and RNA used in this work are presented in Supplementary S1. 

### 3.3. Isothermal Titration Calorimetry

The interaction of Cas9 with nucleic acids was analyzed using the Nano ITC isothermal titration calorimeter (TA Instruments, New Castle, DE, USA). All ITC measurements were carried out at 25 °C with 200 rpm stirring and 300 s delay between successive injections at 1–2 μL using a syringe 50 μL in volume to the sample cell (190 μL). Measurements were run in the overfilled mode to prevent air bubbles, liquid evaporation or the presence of the vapor phase. All experiments were performed in the buffer containing 150 mM NaCl, 20 mM HEPES, pH = 7.6. 

During the key experiment, the cell contained the preassembled Cas9–sgRNA complex with concentrations of 6 μM and 1 μM, respectively. The dsDNA at a concentration of 25 μM was gradually added to the cell. 

In the second experiment, the cell also contained Cas9 (5 μM) in the complex sgRNA (1 μM), to which ssDNA solution (10 μM) was added. 

The thermodynamic parameters of complex formation were determined by adding the sgRNA solution (62.5 μM) to the Cas9 (5 μM) in the cell.

We obtained the parameters of DNA duplex formation by mixing a DNA strand complementary to RNA (20 μM) with a second strand in the cell (2 μM). Additionally, we conducted an experiment by adding a DNA duplex at a concentration of 25 μM to an RNA solution (cell) with a concentration of 10 μM.

We carried out control experiments by adding RNA (50 μM), dsDNA (25 μM), and ssDNA (20 μM) solutions to the buffer in the cell.

The data were processed with the NanoAnalyze software (TA Instruments, New Castle, DE, USA). Models of independent binding or dimer dissociation were applied to determine thermodynamic parameters and stoichiometry of binding.

### 3.4. Computer Simulation

#### 3.4.1. Complexes Containing Cas9

We used the crystal structure of the protein *S. pysogenes* Cas9 (SpCas) with PDB ID 4OO8 as the starting structure of the triple complex of the protein with guide RNA (sgRNA) and ssDNA [12]. The initial structure contained sgRNA (98 nucleotides) and ssDNA (23 nucleotides). The protein structure was modified with Modeller [44], and the nucleic acid sequence was replaced with UCSF Chimera 1.15 [45]. The final model of the complex contained SpCas9 (1368 a.a.), sgRNA (98 nucleotides) and ssDNA (24 nucleotides). The complex of the protein with sgRNA and the structure of the protein itself were obtained by sequential removal of the components from the full-size complex. All structures also contain Mg^2+^ ions, arranged according to [46].

One Mg^2+^ ion was coordinated by the carboxylate moieties of Asp-10, Glu-762 and Glu-766 amino acids. The second Mg^2+^ ion was located next to the first, and coordinated by Asp-10, Glu-766 and His-983 amino acids. Additionally, the third was arranged by His-1297 and Asp-1328 amino acids. The first two Mg^2+^ ions are located in the RuvC domain of the Cas9 protein and play a critical role in the cleavage of the DNA substrate. It is known that Asp-10 is involved in the cleavage of non-complementary DNA strands, and the Mg^2+^ ion is required by Cas9 to ensure enzymatic activity [46].

The ff14SB force field was used [47,48] for the protein and the TIP3P force field for the water molecules, metal ions and counter ions. We used the amber force fields OL3 and bsc1 for the RNA and DNA molecules, respectively [49]. Prior to the molecular dynamics simulation, the energy of the system was minimized in an implicit solvent model using the sander module of Amber20 [50]. After a preliminary preparation step, the system was neutralized with Na^+^ ions and solvated with water molecules using tLEaP. The TIP3P with truncated “cubic” periodic boundary conditions with a distance of 8 Å was used as an explicit water model. Thus, the Cas9–RNA/DNA complex contained 56,490, the Cas9–RNA complex contained 56,705 and the native protein model had 57,282 water molecules.

To reduce undesirable steric interactions and direct the Cas9 contain systems (apo-Cas9, Cas9–RNA, and Cas9–RNA/DNA) to energetically favorable conformations, we used a two-step process of energy minimization by the steepest descent method. In the first step, the solvated elements of the system (complexes and the protein itself) were held at a harmonic force constant of 25 kcal/mol-Å^2^ and water molecules were allowed to relax. In a second step, the restriction was removed to allow all atoms to move freely. Both minimization steps were realized in 1000 cycles. After the energy minimization step, each Cas9-containing system was heated gradually from 1 to 300 K for 125 ps with periodic boundary conditions. The SHAKE algorithm was applied to constrain all bond lengths involving hydrogen to their equilibrium distance. Density equilibration was carried out sequentially for 50 ps and the whole system for 500 ps without constraint. Equilibrium molecular dynamics simulations of 100 ns duration for the Cas9 protein or its complexes with nucleic acids were performed in the NPT ensemble. We analyzed the obtained trajectories using CPPTRAJ [51] and UCSF Chimera [45].

#### 3.4.2. Nucleic Acid Complexes

The starting structures of RNA and the RNA complex with ssDNA were obtained from the initial structure of the Cas9–RNA/DNA. We generated the dsDNA complex using XLEaP. Nucleic acid complexes were pre-minimized, and then neutralized and solvated using tLEaP as described above. Thus, 29,919 molecules of water were added to the RNA/DNA complex, 28,912 to the RNA complex, and 7700 to the dsDNA complex.

We performed the energy minimization, heating, and equilibration steps the same way as for the protein-containing models. The CPPTRAJ and UCSF Chimera were used to analyze molecular dynamics trajectories.

### 3.5. MM-PBSA Calculations

We analyzed the obtained molecular dynamics trajectories using molecular mechanics energies combined with the molecular mechanics energies combined with the Poisson–Boltzmann or generalized born and surface area continuum solvation (MM-PBSA and MM-GBSA) to calculate the thermodynamic parameters of the Cas9–RNA/DNA, Cas9–RNA, RNA/DNA, and DNA/DNA complexes.

While studying the thermodynamic aspects of complex formation, the complex is divided into a so-called “receptor” and “ligand” molecule. The topology files for the components of the complexes being studied were generated before the analysis by CPPTRAJ. The ionic strength of the solution was taken equal to 100 mM of monovalent cations. Each step of the MD trajectory was used in the calculations.

## 4. Conclusions

This study shed light on the first step of DNA processing by CRISPR–Cas systems—formation of the Cas9–RNA/DNA complex. We analyzed the thermodynamics of the interaction of a complex’s components by experimental and computer simulation methods. The in vitro experiments using isothermal titration calorimetry showed the complexity of the studied system. We have found that non-profit nucleic acid interactions complicate the thermodynamic analysis. Nonetheless, we found that there is a small energetic preference during Cas9–RNA/DNA formation from the Cas9–RNA and DNA/DNA duplex. These experimental findings were confirmed by molecular dynamics simulations and analysis of the trajectories via MM-PBSA and MM-GBSA analysis. The small difference in binding energy is relevant for biological interactions and could be part of sequence-specific recognition of the double-stranded DNA CRISPR–Cas9 system. Obtained thermodynamic parameters and K_d_ of Cas9 interactions with nucleic acids could help to model the formation of complexes in solution. Our data open up perspectives for the fine tuning of the CRISPR–Cas9 system based on knowledge of the thermodynamic characteristics of each component of the complex.

## Figures and Tables

**Table 1:** The thermodynamic parameters obtained by ITC.

Cell	Syringe	K_d_, M	*n*	ΔH°, kcal/mol	ΔS°, kcal/mol·K	ΔG°(25 °C), kcal/mol
Substance, μM	Substance, μM					
buffer	RNA, 50 μM	1.62 × 10^−6^	-	−95.25	−346.0	7.90
buffer	RNA, 10 μM	˗	˗	˗	˗	˗
buffer	dsDNA, 25 μM	˗	˗	˗	˗	˗
buffer	ssDNA, 20 μM	˗	˗	˗	˗	˗
RNA,10 μM	dsDNA, 25 μM	˗	˗	˗	˗	˗
RNA, 2 μM	ssDNA, 20 μM	6.36 × 10^−7^ ± 2.43 × 10^−7^	0.11 ± 0.14	−429.2 ± 542.4	−1411	−8.454
ssDNA, 2 μM	ssDNA1, 20 μM	2.09 × 10^−7^ ± 0.67 × 10^−7^	0.83 ± 0.05	−212.2 ± 17.7	−681.1	−9.112
Cas9, 10 μM	ssDNA, 20 μM	˗	˗	˗	˗	˗
Cas9, 5 μM	dsDNA, 25 μM	˗	˗	˗	˗	˗
Cas9 *, 5 μM	RNA, 62.5 μM	2.21 × 10^−6^ ± 0.67 × 10^−6^	2.10 ± 0.12	−399.10 ± 39.12	−1313	−7.72
Cas9, 5 μM RNA, 1 μM	ssDNA, 10 μM	9.80 × 10^−9^ ± 1.87 × 10^−8^	0.67 ± 0.07	−49.27 ± 8.73	−128.60	−10.93
Cas9 **, 6 μM RNA, 1 μM	dsDNA, 25 μM	1.02 × 10^−7^ ± 5.90 × 10^−7^	0.76 ± 0.68	−45.08 ± 302.4	−119.02	−9.54

* in triplicate, ** in duplicate.

**Table 2:** Values of complex formation energies obtained with MM-GBSA and MM-PBSA.

	Receptor	Ligand	MM-GBSA	MM-PBSA
			ΔE, kcal/mol	ΔE, kcal/mol
Cas9–RNA/DNA	Cas9–RNA	ssDNA	−363.9 ± 0.2	−149.0 ± 0.2
Cas9–RNA	Cas9	RNA	−820.0 ± 0.3	−948.2 ± 0.4
RNA/DNA	RNA	ssDNA	−208.7 ± 0.1	6.1 ± 0.1
dsDNA, 32 nt	ssDNA	ssDNA1	−296.3 ± 0.1	−163.7 ± 0.1
dsDNA, 24 nt	ssDNA	ssDNA1	−221.3 ± 0.1	−140.7 ± 0.1

## Data Availability

Data are available on request, owing to privacy and ethical restrictions.

## Supplementary Materials

The following are available online at https://www.mdpi.com/article/10.3390/ijms23168891/s1.
