# Supplementary material for: Thermodynamic Swings: How Ideal Complex of Cas9–RNA/DNA Forms
Source: Int J Mol Sci. 2022 Aug 10;23(16):8891. doi: 10.3390/ijms23168891 (PMC9408429; doi:10.3390/ijms23168891)
Supplement: Supplementary file 1 [file ijms-23-08891-s001.zip › ijms-1811166-supplementary.pdf]

S1

The sequences of all DNA and RNA.

1. ITC experiments.

RNA, 103 nt:

5`-
GGUUGGACAUGCUCGACAUUCGUUUUAGAGCUAGAAAUAGCAAGUUAAAAUAAG
GCUAGUCCGUUAUCAACUUGAAAAAGUGGCACCGAGUCGGUGCUUUUUU-3`

ssDNA, 32 nt:

5`- GATCT*CCC*GAATGTCGAGCATGTCCAACTCAC -3`

ssDNA1, 32 nt:

5`-GTGAGTTGGACATGCTCGACATTCGGGAGATC-3`

2. MD simulations.

RNA, 98 nt:

5`-

GGUUGGACAUGCUCGACAUUCGUUUUAGAGCUAGAAAUAGCAAGUUAAAAUAAG

GCUAGUCCGUUAUCAACUUGAAAAAGUGGCAC-3`

ssDNA, 24 nt:

5`- GAATGTCGAGCATGTCCAACTCAC -3`

ssDNA1, 24 nt:

5`-GTGAGTTGGACATGCTCGACATTC-3`

ssDNA, 32 nt:

5`- GATCT*CCC*GAATGTCGAGCATGTCCAACTCAC -3`

ssDNA1, 32 nt:

5`-GTGAGTTGGACATGCTCGACATTCGGGAGATC-3`

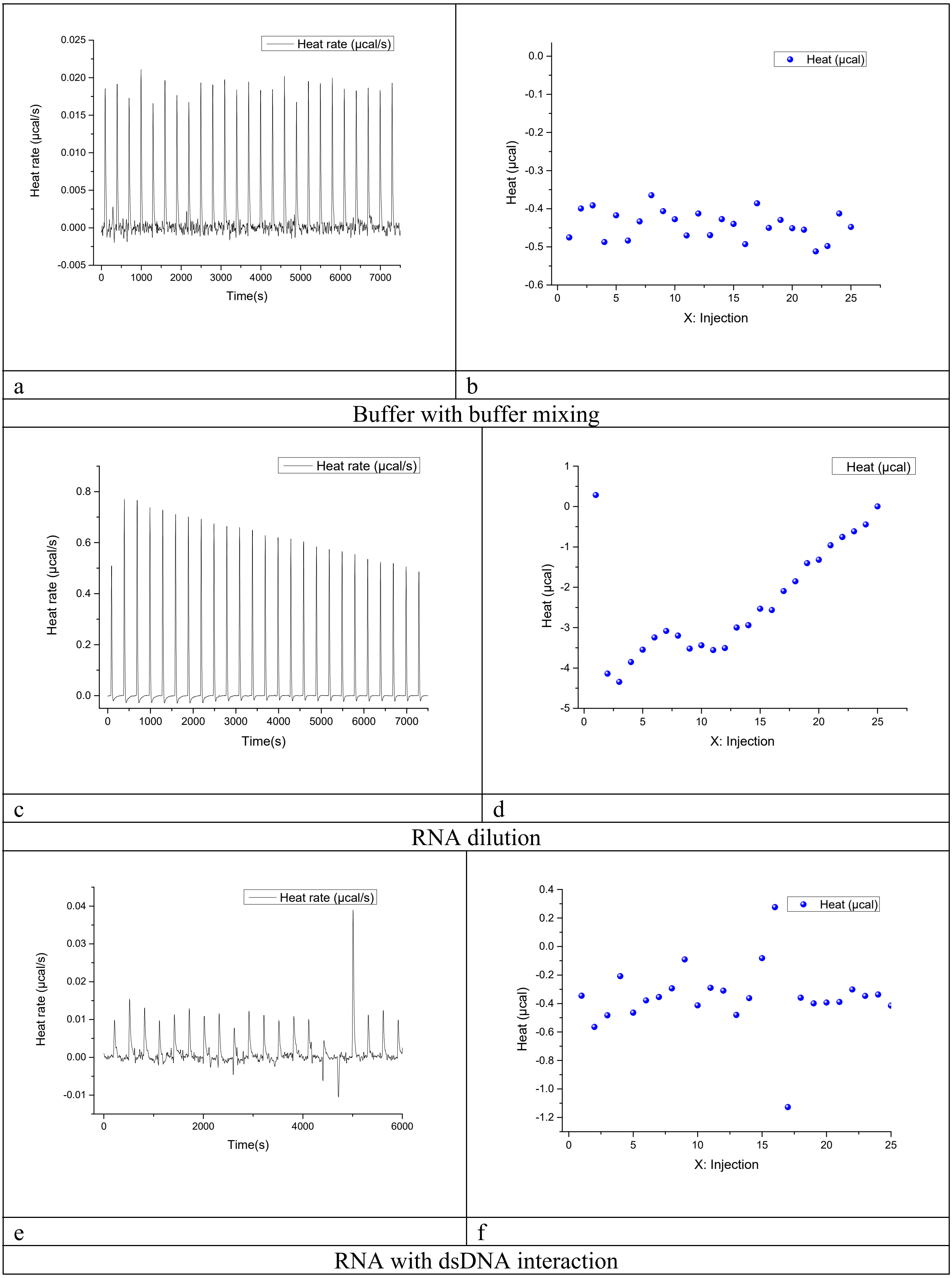
Heat rate (µcal/s)
Heat rate (µcal/s)
Time(s)
Heat (µcal)
Heat (µcal)
X: Injection
a
b
Buffer with buffer mixing
Heat rate (µcal/s)
Heat rate (µcal/s)
Time(s)
Heat (µcal)
Heat (µcal)
X: Injection
c
d
RNA dilution
Heat rate (µcal/s)
Heat rate (µcal/s)
Time(s)
Heat (µcal)
Heat (µcal)
X: Injection
e
f
RNA with dsDNA interaction

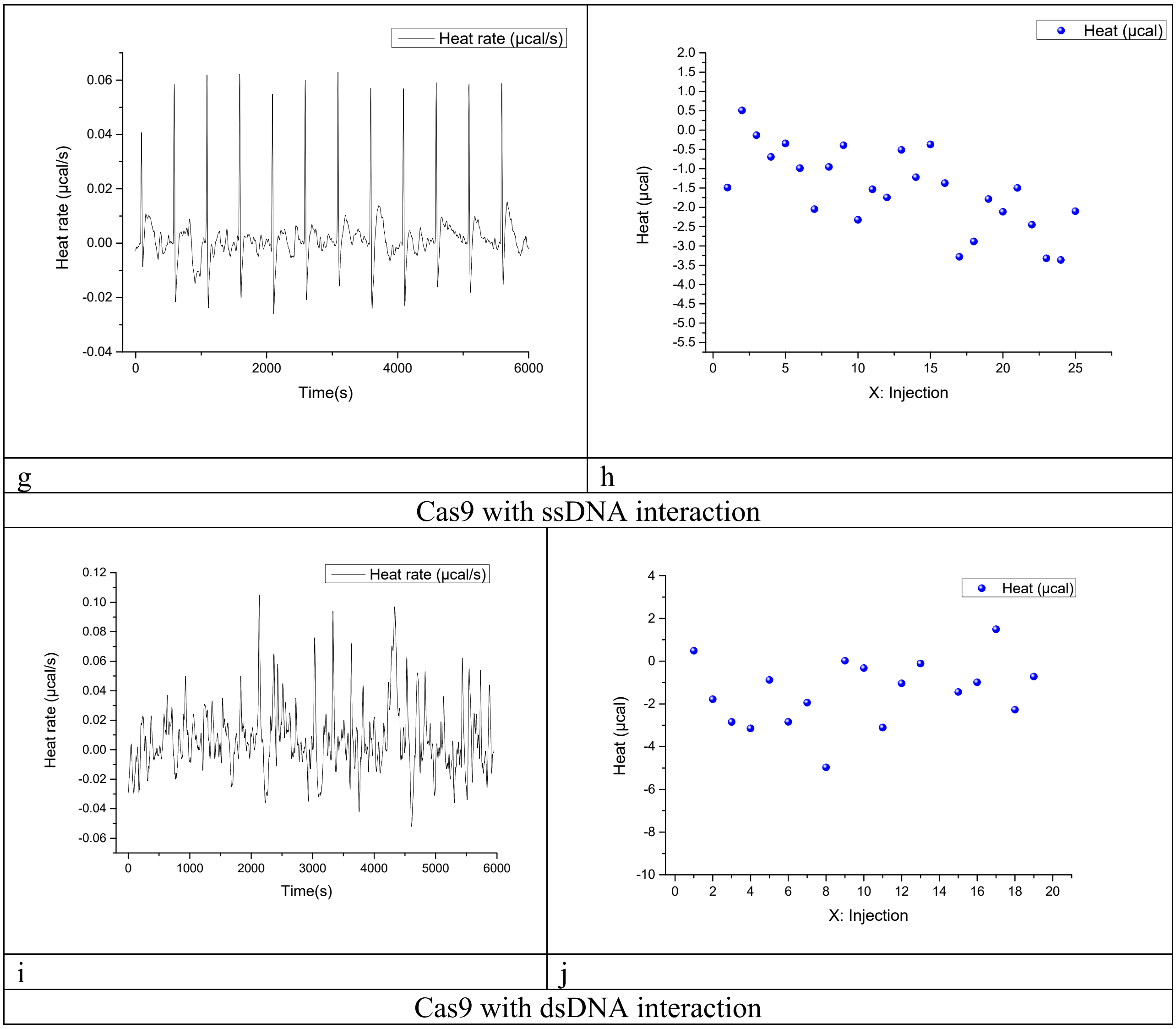

Figure S1. The ITC profiles (A,C,E,G,I) and corresponding integrated areas of each heat burst curve plotted as a function of injection number (B,D,F,H,J) for buffer mixing (A,B), for RNA dissolving (C,D), for interaction of RNA with dsDNA (E,F), Cas9 with ssDNA (G,H) and Cas9 with dsDNA (I,J)

a

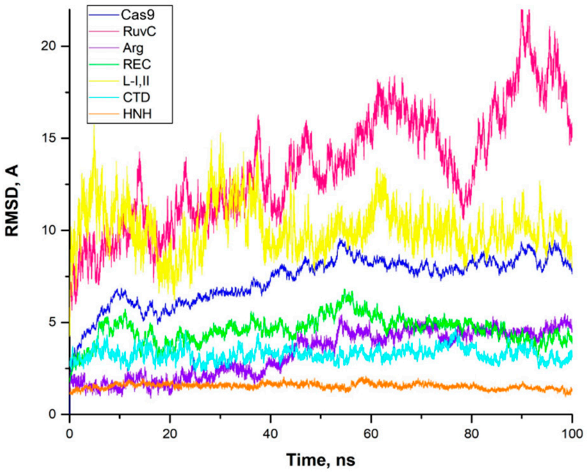

b

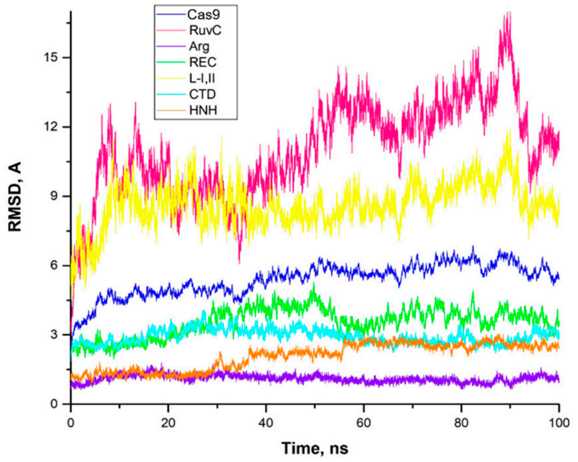

c

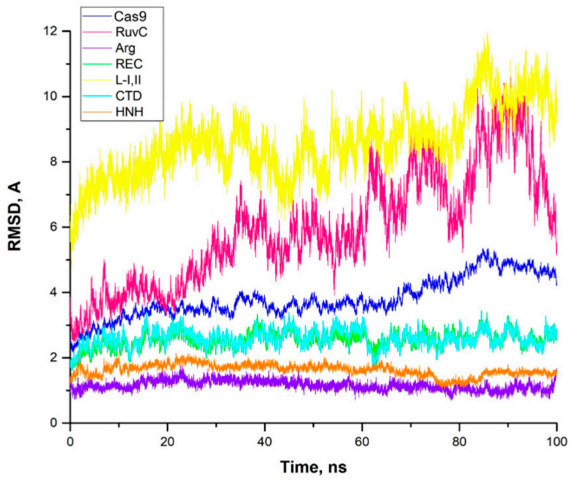

Figure S2. RMSD of Cas9 (blue) and its domains in apoCas9 (**a**), in the Cas9/RNA complex (**b**), and Cas9-RNA/DNA (**c**). The plots were obtained using CPPTRAJ for the entire 100 ns MD trajectories. The RuvC domain is shown in pink, Arg in purple, REC in green, L-I,II in yellow, CTD in blue, and HNH in orange.

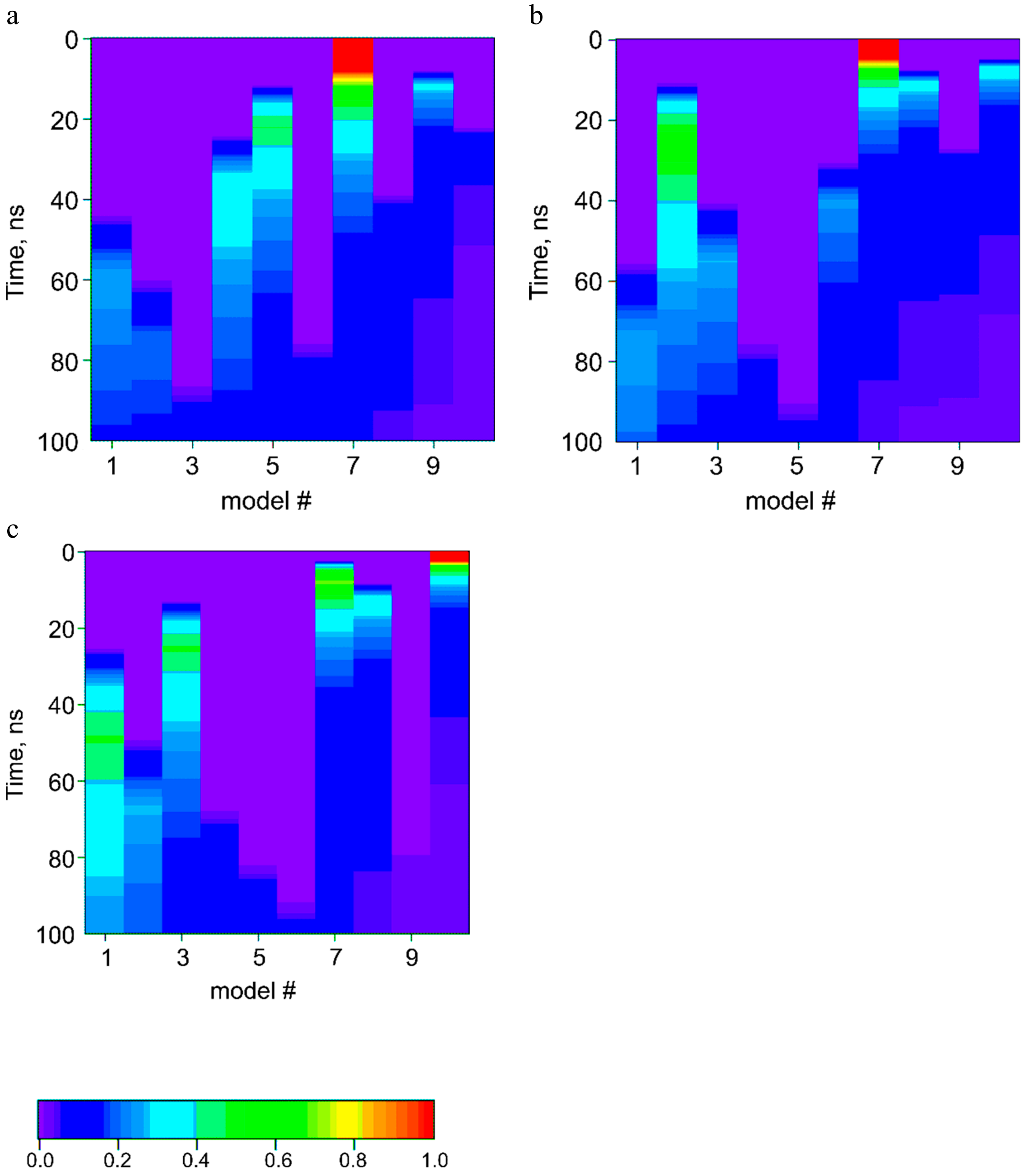

Figure S3 The representation of clusters as a function of dynamics time for apoCas9 (**a**), for its Cas9/RNA (**b**), and Cas9-RNA/DNA (**c**) complexes. The x-axis shows the number of the represented structure (model) from 1 to 10, the y-axis shows the dynamics time in ns, the scale at the bottom reflects the representation from the lowest (blue) to the highest (red).

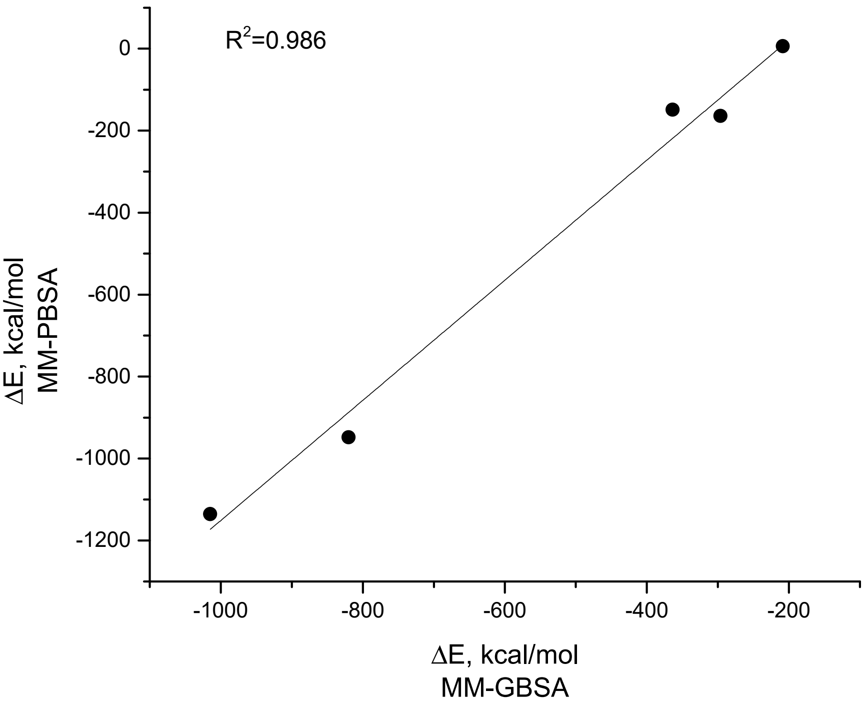

Figure S4. Correlation of the binding energies calculated by the MM-PBSA and MM-GBSA methods. The x-axis shows the energies resulting from the MM-GBSA method in kcal/mol, and the y-axis shows the ΔE obtained from the MM-PBSA trajectory analysis in kcal/mol.
